# 3D Genome Architecture in Stem Cell Lineage Commitment: from Structural Organization to Precision Regulation

**DOI:** 10.1002/ggn2.202500035

**Published:** 2025-09-16

**Authors:** Yanchi He, Wenrui Li, Lin Li, Ying Yang, Yutong Lu, Yufei Pan, Qing Wang, Yuqiang Sun, Yuxuan Xie, Mingyue Wu, Peng Luo, Wansu Sun, Hengguo Zhang

**Affiliations:** ^1^ College & Hospital of Stomatology Anhui Medical University Key Lab. of Oral Diseases Research of Anhui Province Hefei 230032 China; ^2^ Second School of Clinical Medicine Anhui Medical University Hefei 230032 China; ^3^ First School of Clinical Medicine Anhui Medical University Hefei 230032 China; ^4^ Pharmaceutical Sciences Anhui Medical University Hefei 230032 China; ^5^ Department of Oncology Zhujiang Hospital Southern Medical University Guangzhou 510280 China

**Keywords:** 3D genome, epigenetic, lineage commitment, stem cell

## Abstract

Stem cell lineage commitment is governed by intricate interactions between epigenetic mechanisms and 3D genome organization. Traditional linear epigenetics, including DNA methylation and histone modifications, cannot fully elucidate the complex spatiotemporal regulation of gene expression. Recent advances in spatial genomics technologies, such as high‐throughput chromosome conformation capture (Hi‐C), single‐cell Hi‐C, and Chromatin immunoprecipitation combined with Hi‐C (Hi‐ChIP), have provided unprecedented insights into genome architecture, revealing key structural units like chromatin compartments, topologically associating domains (TADs), and chromatin loops. These structures dynamically reorganize during differentiation, influencing transcriptional accessibility and lineage‐specific gene activation. Additionally, liquid‐liquid phase separation (LLPS)‐mediated transcriptional condensates, such as transcription factories and super‐enhancers, have emerged as essential regulators of gene expression patterns during cell fate transitions. The integration of multiomics data and artificial intelligence‐driven predictive modeling further enhances the understanding of these regulatory networks. Despite ongoing technical challenges, including limitations in resolution, data complexity, and causal inference, recent advances continue to push the field forward. Engineered interventions such as CRISPR‐based spatial genome editing and AI‐powered computational platforms hold great promise for translating structural insights into targeted therapeutic strategies in regenerative medicine.

## Graphical abstract

The article explores how the 3D structure of DNA governs stem cell fate. Key architectural elements and advanced tools are described revealing dynamic genome organization. The work links spatial genome changes to precise gene control during cell differentiation. It also addresses current challenges and proposes integrated strategies to translate 3D genomics into clinical applications.

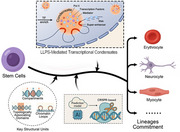

## 1 Introduction

### 1.1 From Linear Epigenetics to Spatial Genomics

**Figure 1:**
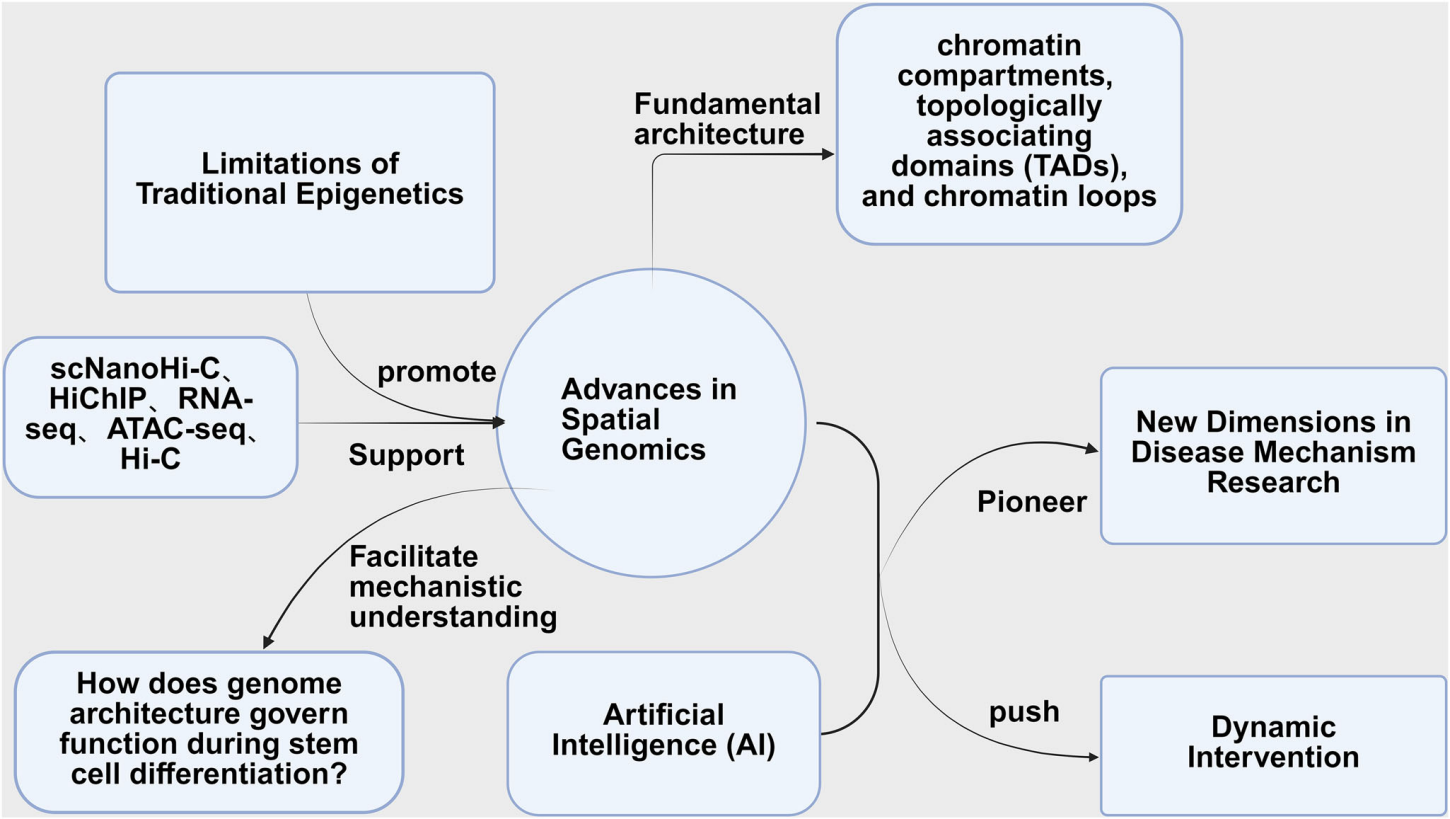
Traditional epigenetics, hindered by its linear analytical approach, struggles to decode gene expression regulation across spatiotemporal dimensions. Advances in scNanoHi‐C, HiChIP, RNA‐seq, ATAC‐seq, and Hi‐C have enabled studies of 3D genome architecture, revealing structures like chromatin compartments, topologically associating domains (TADs), and chromatin loops, addressing gaps in traditional methods. Spatial genomics offers fresh insights into how genome structure governs function during stem cell differentiation and merges with AI to explore disease mechanisms innovatively. This field is evolving from structural mapping to dynamic intervention, paving new paths for regenerative medicine and precision disease therapies.

Stem cell lineage commitment refers to the process by which stem cells gradually lose their multidirectional differentiation potential, ultimately determining lineage differentiation.^[^ ^1^ ^]^ Its essence lies in the precise programming of gene expression through epigenetic regulation under specific spatiotemporal conditions.^[^ ^2^ ^]^ Traditional epigenetics primarily focuses on linear mechanisms, such as DNA methylation and histone modifications.^[^ ^3^ ^]^ They regulate gene expression by altering chromatin structure and reversibly switching genes on or off. However, 1D linear epigenetics struggles to explain the spatiotemporal coordination of gene expression during lineage differentiation (**Figure** **1**). For instance, the differentiation of the neural crest stem cells requires spatial interactions between distant enhancers and promoters. But linear epigenetic signals cannot fully resolve such long‐range regulatory networks.^[^ ^4^ ^]^ Additionally, gene mutations in hematopoietic stem cells drive leukemogenesis through 3D genome restructuring. It inadequately interprets the regulatory roles of 98% of noncoding regions mediated by chromatin spatial folding.^[^ ^5^ ^]^ This realization underscores the inherent limitations of unidimensional epigenetic analysis, necessitating the paradigm shift toward spatially resolved genomic interrogation that has revolutionized modern molecular biology.

**Figure 2:**
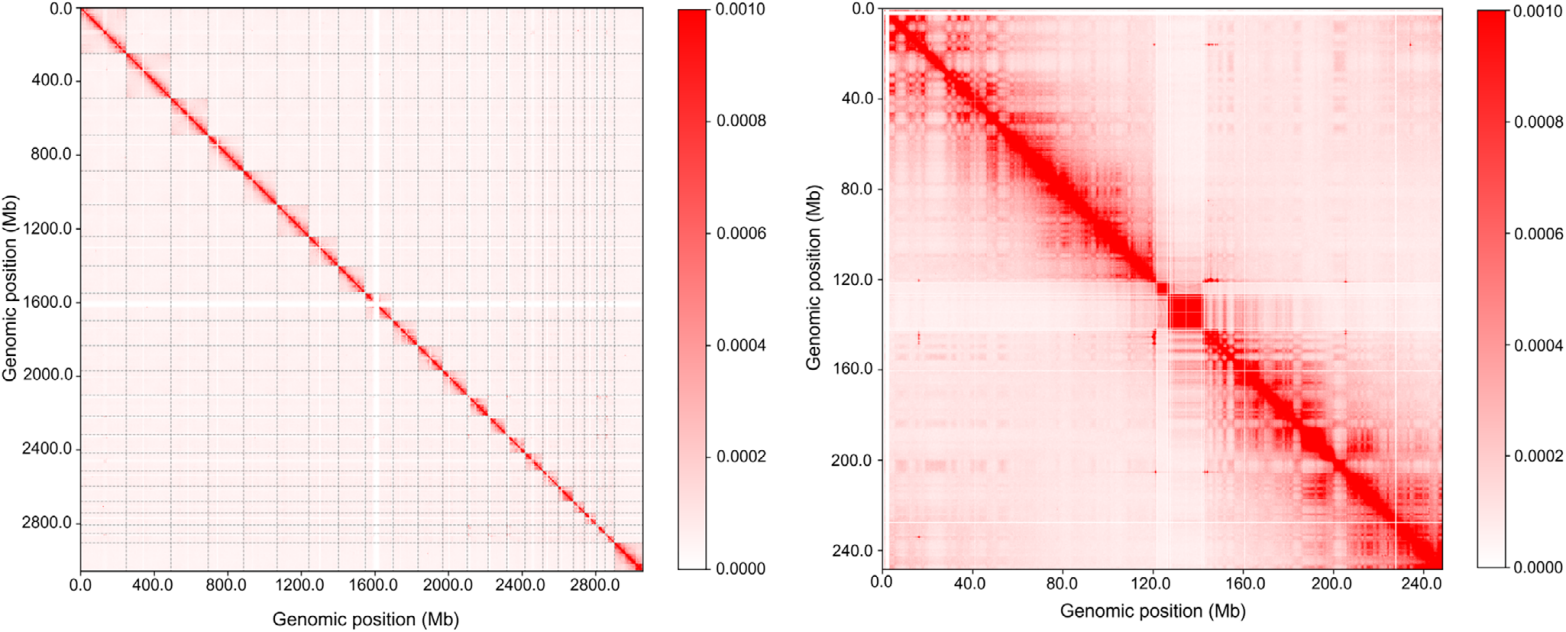
Spatial chromatin architecture across genomic scales (Left) Genome‐wide Hi‐C contact matrix (0–2800 Mb; 1 Mb resolution) reveals long‐range interaction patterns: Diagonal signals reflect linear proximity interactions, while off‐diagonal block‐like structures demonstrate A/B compartmentalization spanning multimegabase scales (transcriptionally active compartments exhibit elevated contact frequencies). Vertical/horizontal gaps indicate heterochromatic regions. (Right) Zoomed‐in view (0–240 Mb; 50 kb resolution) resolves short‐range interaction features: Diagonal stripes demarcate intra‐domain contacts within topologically associating domains (TADs), discrete off‐diagonal points capture specific short‐range interactions mediated by chromatin loops (e.g., enhancer‐promoter contacts), and stripe gaps mark TAD boundaries (insulation sites). Both matrices share a normalized contact frequency scale (0.0000 [blue, low] – 0.0008 [red, high]).

Spatial genomics shifts the perspective from linear epigenetics to viewing the genome as a dynamically folded spatial network. Its foundational architecture comprises three critical structures: chromatin compartments, topologically associating domains (TADs), and chromatin loops (Figure 1).^[^ ^6^ ^]^ Chromatin compartments refer to the division of chromatin into distinct nuclear regions with differing functional properties, primarily categorized as A and B compartments.^[^ ^7^, ^8^ ^]^ Compartment A typically exhibits higher transcriptional activity and is considered an open chromatin region. Whereas Compartment B shows lower transcriptional activity and is associated with gene silencing and heterochromatin formation.^[^ ^9^ ^]^ TADs are structural chromatin units whose boundaries are maintained by regulatory elements such as CTCF proteins and noncoding RNAs.^[^ ^10^ ^]^ Within TADs, chromatin fragments interact closely, forming independent structural units.^[^ ^11^ ^]^ Chromatin loops are regions of high interaction frequency, further subdivided within TADs. It includes long‐range chromosomal interactions (e.g., loops between promoters and distant enhancers) and short‐range chromosomal interactions.^[^ ^9^ ^]^ The rise of spatial genomics has been propelled by technological breakthroughs, enabling epigenetics to transition from planar to 3D studies. Advances in Hi‐C (high‐throughput/high‐resolution chromatin conformation capture), Hi‐ChIP (in situ Hi‐C combined with chromatin immunoprecipitation), and single‐cell Hi‐C have unveiled the dynamic folding patterns of genomes (Figure 1)(**Figure** **2**).^[^ ^7^, ^12^, ^13^ ^]^ For example, using sub‐kb resolution in situ Hi‐C in Drosophila, researchers identified nearly eight times more TADs than previously reported.^[^ ^14^ ^]^ The development of scNanoHi‐C allows effective analysis of 3D chromatin structures and distinguishes structural subtypes among individual cells.^[^ ^15^ ^]^ And it offers new opportunities for studying higher‐order 3D genome organization at the single‐cell level.^[^ ^15^ ^]^

By integrating Hi‐C, ATAC‐seq, and RNA‐seq, copy number variations(CNVs) in multiple myeloma increase TAD numbers by 25%. With breakpoints significantly overlapping TAD boundaries, this suggests that genomic variations interact with 3D structures to drive cancer progression (Figure 1).^[^ ^5^ ^]^ By combining single‐cell 3D genome (scHi‐C), epigenetic modifications (ATAC‐seq, ChIP‐seq), and protein interaction (AP‐MS) data, researchers can construct “digital twin” models of stem cell differentiation. These models simulate chromatin compartment reorganization, transcriptional condensate formation, and predict intervention effects on differentiation pathways.^[^ ^16^ ^]^ These pathways include Lineage Commitment Pathways, Differentiation Blockade Pathways and Differentiation Drift Correction Pathways.^[^ ^17^, ^18^, ^19^ ^]^ Simultaneously, RNA epigenetics adds a new regulatory layer: poly(A) tail dynamics and m6A modifications encode epigenetic information. This encoding occurs through modulating mRNA nucleocytoplasmic transport and spatial localization, refining spatiotemporal precision in stem cell fate decisions. This discovery lays the groundwork for RNA‐centric 3D genome editing tools.^[^ ^20^ ^]^ Recent studies propose that, beyond static structural partitioning, the 3D genome hosts dynamic transcriptional condensates.^[^ ^21^ ^]^ These phase‐separated hubs concentrate transcription machinery and regulatory elements, providing a new layer of control during stem cell fate decisions, particularly under external stimuli or differentiation cues.^[^ ^22^ ^]^

Recently, spatial genomics, integrated with artificial intelligence (AI), has opened new dimensions for disease mechanism research, accelerating medical advancements. AI‐driven 3D structure prediction offers novel tools for deciphering chromatin folding dynamics (Figure 1).^[^ ^23^ ^]^ Deep learning‐based models simulate chromatin loop formation and TAD boundary reconfiguration during stem cell differentiation or carcinogenesis. These models enhance target discovery efficiency by three to fivefold.^[^ ^24^ ^]^ Moving forward, AI‐powered cross‐scale, multimodal platforms will propel 3D genomics from structural resolution to dynamic intervention. This transition pioneers new frontiers in regenerative medicine (Figure 1).

Thus, spatial genomics provides a comprehensive, multidimensional approach to understanding how genome structure governs function during stem cell differentiation (Figure 1). Future integration with artificial intelligence and programmable genome‐editing tools promises to redefine the frontier of developmental biology and regenerative medicine.

## 2 Spatial Genome Architecture in Pluripotency and Lineage Commitment

### 2.1 Static Architecture Supporting Pluripotency

**Figure 3:**
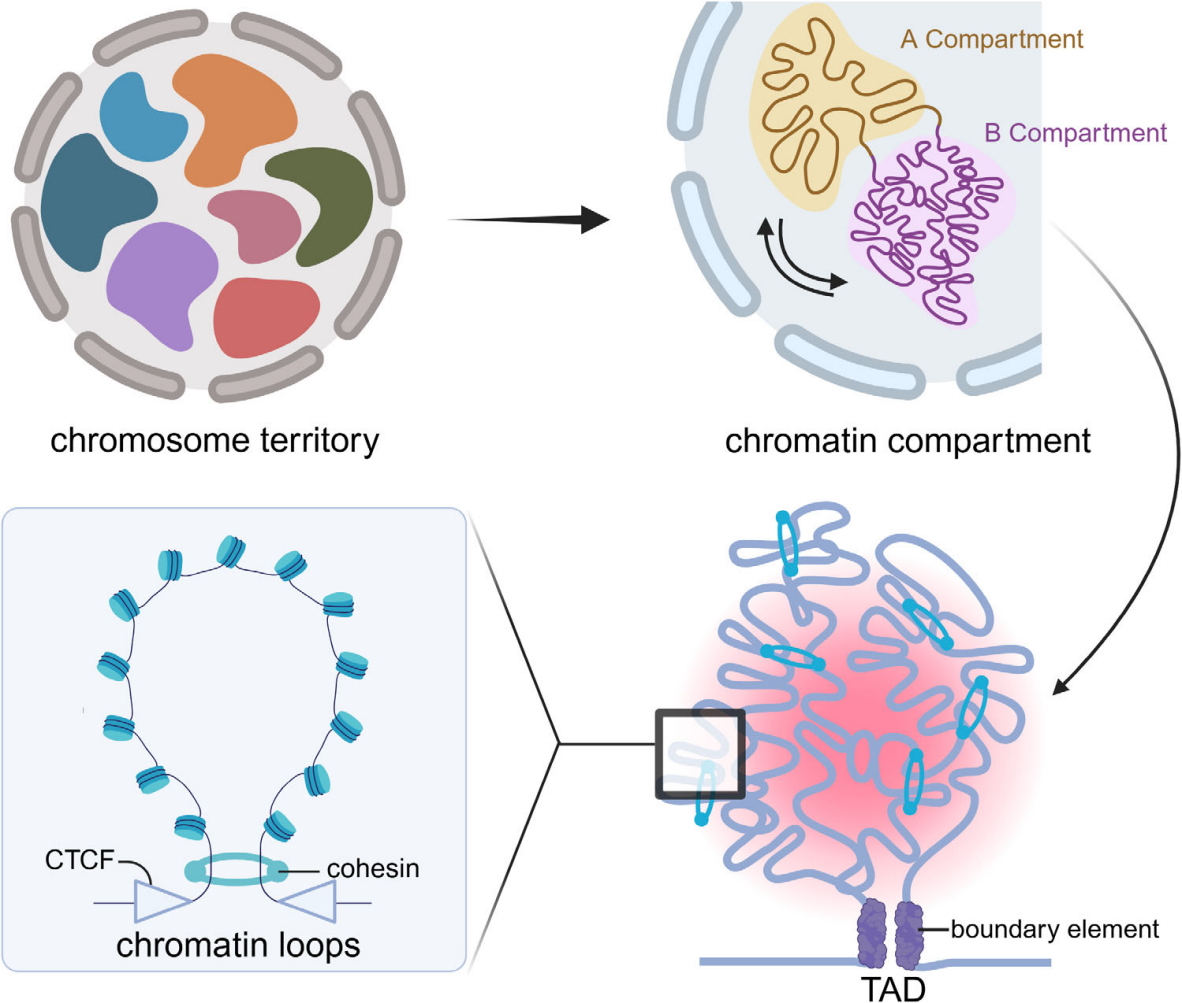
Hierarchical organization of the 3D genome. This diagram shows the static architecture supporting pluripotency in the spatial genome and the relationships between them, including chromatin territories (CTs), chromatin compartments (A/B compartments), topologically associated domains (TADs), and chromatin loops. Created in BioRender. He, J. (2025) https://BioRender.com/tvgv0ez.

The maintenance of pluripotency in stem cells depends on specific 3D genomic structural motifs, including chromosome territory (CT), chromatin compartments, topologically associating domains (TADs), and chromatin loops^[^ ^25^, ^26^ ^]^ (**Figure** **3**). These static architectures work together to establish a unique nuclear environment that enables the pluripotent state.

#### 2.1.1 Chromosome Territory (CT)

Chromosome territories refer to the regions in the nucleus that each chromosome occupies in a specific way.^[^ ^27^ ^]^ These regions exhibit different morphologies and functions at different stages of the cell cycle. Chromosome territories present a highly ordered hierarchical structure in the cell nucleus, which is able to maintain the stability and order of the genome. They occupy a specific spatial location within the nucleus and have certain interactions and exchanges with other chromosome territories.

#### 2.1.2 Chromatin Compartments

Through Hi‐C technology, it was discovered that the entire genome is partitioned into compartments A/B.^[^ ^26^ ^]^ The compartments A are transcriptionally active, enriched in open chromatin, gene‐dense, and have high GC content. They are typically located in the inner part of the nucleus and associated with ongoing transcription and a more open chromatin conformation.^[^ ^26^ ^]^ The compartments B are transcriptionally inert regions associated with closed chromatin, gene‐poor, and compact.^[^ ^26^ ^]^ They are usually situated at the periphery of the nucleus and are characterized by a more condensed chromatin state^[^ ^6^, ^8^ ^]^(Figure 3). Notably, in hematopoietic stem cells compared to differentiated cells, the partitioning between active and inactive compartments is less defined, allowing for greater transcriptional plasticity.^[^ ^28^ ^]^ This plasticity is crucial for the self‐renewal and differentiation potential of pluripotent stem cells.

#### 2.1.3 Topologically Associating Domains

Topologically associated domains (TADs) are the basic structural units of 3D genome organization. Within TADs, chromatin segments interact with each other more frequently than with chromatin segments in other TAD regions.^[^ ^6^, ^8^ ^]^ This higher‐order chromatin organization allows genes within the same TAD to be co‐regulated by shared regulatory elements, such as enhancers and silencers. As a result, gene expression within TADs is relatively independent between different TADs. This compartmentalization of the genome into TADs ensures the precise regulation of key pluripotency genes, such as OCT4, SOX2, and NANOG.^[^ ^6^, ^29^ ^]^ (**Table** **1**) These genes are essential for maintaining the pluripotent state, and their improper regulation can lead to loss of pluripotency and cellular differentiation. TAD boundaries are often demarcated by specific DNA sequence motifs and are maintained by architectural proteins such as CTCF and cohesin.^[^ ^6^, ^30^, ^31^ ^]^ The integrity of TADs is crucial for the proper functioning of the genome, and any alterations in TAD structure can have profound effects on gene regulation and cellular function.^[^ ^8^, ^32^ ^]^

**Table 1:** Key mechanisms regulating the spatial architecture of pluripotency genes (such as OCT4, SOX2, and NANOG).

Section	Specific example	Core biological mechanism
2.1.3 TAD	**OCT4/SOX2/NANOG gene cluster** Located within the same TAD, sharing regulatory elements (e.g., enhancers).	**Intra‐TAD co‐regulation** TAD boundaries (maintained by CTCF/cohesin) insulate pluripotency genes, ensuring precise co‐expression.
2.2.1 Compartment Switching	**Nanog gene** Switches from transcriptionally active compartment A to silenced compartment B during ESC differentiation into neural progenitors.	**Compartment switching(A → B)** Silences pluripotency genes via chromatin condensation, driving lineage commitment.
2.2.2 TAD Boundary Remodeling	**Sox9‐Kcnj2 locus (contrasted with pluripotency gene regulation)** New TAD boundaries form during differentiation to insulate lineage‐specific enhancers.	**De novo TAD boundary establishment** CTCF/cohesin‐mediated insulation prevents ectopic activation of lineage genes by pluripotency regulatory elements.
2.2.3 Chromatin Loop Formation	**Dynamic chromatin loops in pluripotency** CTCF/cohesin‐mediated loops connect enhancers and promoters of pluripotency genes.	**Chromatin loop reorganization** Rapid response to differentiation signals by remodeling pluripotency gene networks (e.g., dissociation of OCT4/NANOG enhancer‐promoter loops).
2.3 Epigenetic Crosstalk	**Compartment A localization of pluripotency genes** (e.g., OCT4/NANOG) enriched with activating marks.	**Bidirectional chromatin structure‐epigenetics feedback** Open compartment A promotes activating modifications, which in turn stabilize compartment positioning to maintain pluripotency.
2.4.2 Super‐Enhancer Condensates	**Nanog super‐enhancer** Phase separation (LLPS) aggregates enhancer elements in pluripotency, enabling synergistic transcriptional activation.	**LLPS‐driven enhancer clustering** IDR‐mediated condensation of transcription factors (e.g., BRD4/Mediator) enhances expression efficiency of pluripotency genes.

#### 2.1.4 Chromatin Loops

Chromatin loops are formed by the interaction between distant chromatin regions in 3D space.^[^ ^28^ ^]^ CTCF is a transcription factor that is enriched at the boundaries of chromatin loops. It can interact with the cohesin complex, a cyclic protein complex, to facilitate the formation and maintenance of chromatin loop structures.^[^ ^33^ ^]^ Chromatin loops bring regulatory elements, such as enhancers and promoters, into close proximity in three dimensions (Figure 2). This enables efficient communication between regulatory elements and target genes, allowing for the precise regulation of gene expression.^[^ ^6^, ^8^ ^]^ In pluripotent stem cells, this 3D genomic organization enables them to rapidly respond to external signals and initiate differentiation programs.^[^ ^28^ ^]^ The dynamic nature of chromatin loops allows for the rapid reconfiguration of gene regulatory networks in response to developmental cues and signaling pathways.^[^ ^34^, ^35^ ^]^ For example, during differentiation, the formation of new chromatin loops can bring lineage‐specific regulatory elements into contact with target genes, activating their expression and driving cellular specialization.^[^ ^36^ ^]^ The CTCF/cohesin‐mediated chromatin loop extrusion model has been proposed to explain the formation of chromatin loops and their role in gene regulation.^[^ ^37^ ^]^ This model suggests that cohesin complexes encircle chromatin fibers and move along them, extruding loops until they encounter CTCF‐bound boundaries.^[^ ^32^ ^]^ This process helps establish the intricate network of chromatin loops that underlie the complex gene regulatory landscape in pluripotent stem cells and during development.

### 2.2 Dynamic Rewiring During Lineage Commitment

#### 2.2.1 Compartment Switching (A to B or Vice Versa) Alters Transcriptional Accessibility

Compartment switches involve particular chromatin regions moving from spatial interactions with euchromatin to heterochromatin interactions, or vice versa. Euchromatin is associated with active transcription, whereas heterochromatin is associated with transcriptional repression.^[^ ^32^ ^]^ This dynamic process can alter transcriptional accessibility and affect gene expression. During cell differentiation, certain regions may transition from an active compartment A to a transcriptionally inert compartment B, or vice versa. For example, in studies of the differentiation of mouse embryonic stem cells into neural progenitor cells, the loci of genes associated with neuronal function (such as Syn1) transition from compartment B to compartment A, accompanied by increased chromatin accessibility and transcriptional activation; in contrast, some pluripotency‐related genes (such as Nanog) undergo the opposite transition (from A to B), accompanied by their silencing^[^ ^28^, ^38^ ^]^ (Table 1). Such compartment switching can be driven by changes in chromatin states, such as DNA methylation, histone modifications, and chromatin accessibility^[^ ^38^ ^]^(Figure 3). These changes can be caused by various factors, including the recruitment of chromatin remodeling complexes and the action of transcription factors.^[^ ^39^, ^40^ ^]^ The repositioning of genes between compartments can influence their transcriptional activity, thereby influencing cell differentiation.

#### 2.2.2 New TAD Boundaries Emerge, Insulating Lineage‐Specific Regulatory Programs

During cell differentiation and development, new TAD boundaries emerge, thereby altering the genomic landscape and gene regulatory networks.^[^ ^41^ ^]^ The emergence of new TAD boundaries helps to segregate the regulatory programs of a particular lineage, ensuring coordinated gene expression within the TAD and insulating it from the influence of regulatory factors in neighboring regions.^[^ ^10^, ^42^, ^43^ ^]^ For example, during mouse neural development, the Sox9‐Kcnj2 gene locus region forms new TAD boundaries. This boundary isolates the neuro‐specific enhancer of Sox9 (a key gene in neural crest development) from the adjacent Kcnj2 gene (a potassium ion channel gene highly expressed in the heart). This new TAD structure ensures that the Sox9 enhancer activates only its own promoter without interfering with Kcnj2, thereby precisely coordinating the differentiation program of the neural lineage while suppressing ectopic activation of unrelated genes.^[^ ^28^, ^44^ ^]^ The establishment and maintenance of TAD boundaries is dependent on a variety of factors, including CTCF‐binding sites, adhesion protein complexes, and chromatin modifications^[^ ^45^, ^46^ ^]^ (Table 1). Disruption of TAD boundaries or their regulatory elements may lead to aberrant gene expression and has been associated with a wide range of disorders. This spatial rewiring is essential for establishing lineage fidelity.^[^ ^31^, ^47^, ^48^ ^]^

#### 2.2.3 De Novo Loop Formation Connects Lineage‐Specific Enhancers and Promoters

Newly formed chromatin loops can bring together lineage‐specific enhancers and promoters that are distant from each other in the linear genome sequence but need to interact for the precise regulation of gene expression.^[^ ^49^ ^]^ In the hematopoietic lineage, the formation of new loops connects promoters and their distal lineage‐specific enhancers of key genes like GATA1 and RUNX1.^[^ ^50^ ^]^ GATA1 is a transcription factor essential for erythroid and megakaryocytic development, while RUNX1 plays a crucial role in hematopoietic stem cell development and differentiation.^[^ ^51^ ^]^ The establishment of these specific enhancer‐promoter loops ensures the timely and appropriate expression of lineage‐determining genes, driving the differentiation of hematopoietic stem cells into various blood cell types^[^ ^50^, ^52^ ^]^ (Table 1). This process involves the coordinated action of multiple chromatin‐associated proteins, such as transcription factors and architectural proteins like CTCF and cohesin.^[^ ^51^, ^53^ ^]^ The dynamic regulation of chromatin looping is essential for the proper execution of lineage‐specific gene expression programs and cellular differentiation.^[^ ^54^, ^55^ ^]^

### 2.3 Functional Crosstalk with Epigenetic Modifications

The 3D genome structure and epigenetic modifications are intricately linked and engage in bidirectional crosstalk to regulate stem cell fate. On the one hand, the spatial organization of the genome influences the accessibility of chromatin to epigenetic regulators. For instance, the positioning of genes within compartments A/B and TADs determines their exposure to histone‐modifying enzymes and DNA methyltransferases. Genes in compartment A are more likely to acquire activating histone modifications, while genes located in compartment B are usually labelled by repressive modifications.^[^ ^50^ ^]^ (Table 1) On the other hand, epigenetic modifications can also affect 3D genome architecture. DNA methylation patterns and histone posttranslational modifications can alter the affinity of chromatin for architectural proteins such as CTCF and cohesin, thereby affecting the formation and stability of TADs and chromatin loops. 3D genomic structure and epigenetics take control of the process of stem cell differentiation together and ensure the successful completion of the stem cell differentiation process.

### 2.4 Phase Separation and Transcriptional Condensates

#### 2.4.1 Transcription Factories: Localized Hubs of RNA Polymerase II and Transcription Factors

Transcription factories are localized centers formed by the enrichment of RNA polymerase II and transcription factors in the nucleus. Liquid‐liquid phase separation (LLPS) plays an important role in its formation.^[^ ^56^, ^57^ ^]^ This mechanism causes aggregation of RNA polymerase II, which is widely distributed in the nucleus, to form a transcription factory.^[^ ^58^, ^59^ ^]^ During this process, RNA polymerase II interacts with other transcription factors, cofactors, and DNA‐binding proteins to form highly ordered and dynamic condensates. These condensates provide a large number of weak, synergistic interaction sites through multivalent interactions (e.g., multistructural domain interactions of transcription factors, multibinding site collaborations of DNA, and crucially, the multivalent, low‐affinity interactions mediated by the specific amino acid sequences (grammar) within intrinsically disordered regions (IDRs) of these components), which enable efficient aggregation of related molecules.^[^ ^22^, ^57^ ^]^ This phase‐separation process enhances the local concentration of transcription factors and RNA polymerase II. At the same time, it enhances their functional interactions, creating favorable conditions for the efficient initiation and continuation of the transcription process. Transcription processes within transcription factories are highly active, and multiple genes can be transcribed simultaneously, greatly enhancing the efficiency of gene expression.^[^ ^58^, ^60^ ^]^ Each transcription factory typically contains multiple active genes that are located in close proximity to RNA polymerase II and other transcription factors in a looped or folded form, creating a highly organized transcription environment.^[^ ^22^ ^]^ In addition, transcription factories are dynamic, capable of rapidly adjusting their composition and location in response to cellular demands. During cellular stress or differentiation, a transcription factory can rapidly change the combination of genes and transcription factors it contains to adapt to new transcription tasks.^[^ ^61^ ^]^ This dynamic adjustment capability allows it to respond flexibly to changes in the intra‐ and extracellular environments and ensures precise regulation of gene expression.^[^ ^56^ ^]^ For example, in Drosophila embryonic development, the composition and location of transcription factories change at different stages, affecting the precision of gene expression.^[^ ^62^ ^]^ At the early embryonic development stage, the transcription factories are enriched with maternally derived transcription factors, which precisely regulate early gene expression. As the embryo develops, the syncytial genes are gradually activated, and the proportion of syncytial transcription factors in the transcription factories increases, ensuring the accurate expression of syncytial genes.^[^ ^61^ ^]^

#### 2.4.2 Super‐Enhancer Condensates: LLPS‐Mediated Clustering of Enhancers Boosts Expression of Cell Identity Genes

A super‐enhancer cohesion is a structure formed by the aggregation of enhancers mediated by liquid‐liquid phase separation (LLPS).^[^ ^21^ ^]^ A super‐enhancer is a segment of DNA sequence in the genome that strongly activates gene expression, usually associated with a cellular identity gene. The formation of these cohesions allows multiple enhancers to come together and enhance the activity of the promoter with which they are associated, thereby boosting the expression levels of cell identity genes.^[^ ^63^ ^]^ The formation of hyper‐enhancer cohesions relies on the synergistic action of multiple transcription factors and cofactors that often contain extensive intrinsically disordered regions (IDRs). These IDRs, characterized by specific amino acid compositions and motifs (their “grammar”) that favor weak, multivalent interactions, are critical drivers of LLPS, enabling the assembly of dynamic condensates. For example, certain transcription factors such as the Mediator complex and bromodomain proteins (e.g., BRD4, whose large IDRs are rich in tyrosine and other residues conducive to phase separation) play key roles in cohesion formation^[^ ^63^ ^]^ (Table 1). These factors form a highly ordered network structure by binding to specific sequences on the enhancer and interacting with other transcription factors. This structure not only improves the efficiency of the interaction between enhancers and promoters but also facilitates the recruitment of RNA polymerase II and the assembly of the transcription initiation complex.^[^ ^64^ ^]^ The super‐enhancer condensate plays a crucial role in cell differentiation and development. In the differentiation of mouse embryonic stem cells into neural cells, the super‐enhancer driving Neurod1 expression contains multiple closely arranged enhancer elements (such as hs158, hs230, etc.). These elements aggregate through phase separation‐mediated condensate formation, greatly enhancing their synergistic activation of the Neurod1 promoter.^[^ ^21^, ^63^ ^]^ The super‐enhancer of Sox9 also exhibits similar characteristics and dynamic recombination.^[^ ^44^ ^]^

#### 2.4.3 Dynamics of Cohesion: Regulating the Landscape of Gene Expression During Differentiation

The dynamic formation or dissolution of transcriptional cohesions (including transcription factories and super‐enhancer cohesions) plays a key role in shaping the gene expression landscape during cell differentiation.^[^ ^65^ ^]^ The formation and depolymerization of these cohesions are highly dynamic, enabling rapid adjustment of gene expression patterns in response to the demands of cell differentiation. It has been shown that the dynamics of cohesion are closely related to the activation of intracellular signaling pathways. Under the stimulation of differentiation signals, specific transcription factors and cofactors, many harboring IDRs with phase‐separation‐prone amino acid grammars rapidly aggregate through the phase separation mechanism to form cohesion and activate the expression of specific genes. When the cell differentiates to the next stage, the condensate dissolves again, releasing the molecules so that they can be redistributed to other regions to participate in the regulation of new gene expression.^[^ ^21^ ^]^ This reversibility is fundamentally linked to the biophysical nature of IDRs: their inherent flexibility and the tunable strength of their multivalent interactions, dictated by amino acid sequence and post‐translational modifications, allow condensates to rapidly assemble and disassemble in response to cellular cues. For example, during the early stages of hematopoietic stem cell differentiation into the erythroid lineage, the formation of GATA1 super‐enhancer clusters promotes the expression of GATA1 itself and its downstream erythroid genes, thereby initiating the erythroid differentiation program.^[^ ^21^ ^]^ As differentiation advances, these cohesions gradually depolymerize to make way for the regulation of gene expression at subsequent stages.^[^ ^21^ ^]^ This dynamic change not only ensures precise and coordinated gene expression during differentiation, but also provides flexibility for cells to adapt to environmental changes. In addition, dynamic regulation of the cohesion involves a variety of epigenetic modifications and chromatin remodeling processes. For example, histone modifying enzymes and chromatin remodeling complexes can influence cohesion formation and stability by altering the state of chromatin.^[^ ^66^ ^]^ This multilevel regulatory mechanism enables fine regulation of gene expression during the complex differentiation process and ensures that cells differentiate and develop according to a predetermined program.

## 3 Current Challenges and Unresolved Questions

### 3.1 Technological Limitations

#### 3.1.1 Complex Data Analysis Pipelines and Large‐Scale Data Handling

**Figure 4:**
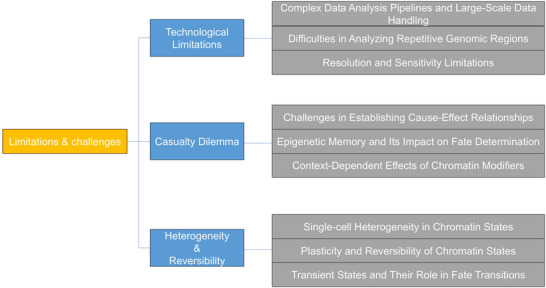
A concise overview of major obstacles in chromatin dynamics studies. Pipeline: The standardized computational workflow for processing raw sequencing data into interpretable results. Heterogeneity: Cell‐to‐cell variations in chromatin states. Reversibility: The dynamic rewinding of chromatin states during fate transitions. Plasticity: Chromatin's adaptive capacity to remodel rapidly in response to environmental cues.

The analysis of 3D genome architecture presents unprecedented computational challenges due to the enormous datasets generated by current technologies (**Figure** **4**). Hi‐C and related chromosome conformation capture methods routinely produce billions of sequencing reads that must undergo multiple processing steps, including alignment, filtering, normalization.^[^ ^67^, ^68^ ^]^ Each of these computational steps introduces potential biases and artifacts that can significantly affect downstream analysis, requiring researchers to implement rigorous quality control measures at every stage of data processing. The massive computational resources needed for processing these datasets, including high‐performance computing clusters with substantial memory capacity, create a significant bottleneck in 3D genome studies.^[^ ^69^, ^70^ ^]^ This resource limitation becomes particularly acute when studying dynamic processes like stem cell differentiation that require multiple time points or biological replicates.

The integration of multiomics data with 3D genome information presents additional layers of analytical complexity that current computational approaches are still struggling to address effectively. Combining Hi‐C data with complementary datasets such as chromatin accessibility (ATAC‐seq), histone modification patterns requires specialized algorithms and statistical methods to identify biologically meaningful patterns and relationships.^[^ ^68^, ^71^ ^]^ Many existing bioinformatics tools were developed for analyzing single data types and lack the sophistication needed to properly integrate information across multiple omics layers, particularly when studying dynamic changes during critical developmental transitions like stem cell lineage commitment.^[^ ^72^, ^73^ ^]^ The field currently suffers from a lack of standardized protocols and benchmarks for data processing and integration, making comparisons between studies from different laboratories difficult and potentially limiting the reproducibility of findings.^[^ ^74^, ^75^ ^]^ This standardization gap represents a significant obstacle to progress in understanding how 3D genome organization contributes to cell fate decisions.

Recent advances in single‐cell 3D genome technologies have dramatically increased the complexity of data analysis pipelines while providing unprecedented resolution of cellular heterogeneity in stem cell populations. Single‐cell Hi‐C methods, while powerful for revealing cell‐to‐cell variation in chromatin architecture, generate data that is extremely sparse and noisy compared to traditional bulk approaches.^[^ ^76^, ^77^ ^]^ Specialized statistical methods and machine learning approaches are required to distinguish technical artifacts from genuine biological variation, particularly when studying rare stem cell subpopulations or transient intermediate states during differentiation.^[^ ^78^, ^79^ ^]^ The computational challenges are further compounded when attempting to correlate single‐cell chromatin conformation data with other single‐cell modalities like transcriptomics or epigenetics. Despite these difficulties, the development of more user‐friendly analysis platforms and visualization tools remains an urgent need to make these powerful but complex technologies accessible to biologists without specialized computational expertise.^[^ ^80^, ^81^ ^]^ Current efforts to create standardized workflows and benchmarking datasets should help address some of these challenges, but significant work remains to fully realize the potential of single‐cell 3D genome analysis in stem cell research.

#### 3.1.2 Difficulties in Analyzing Repetitive Genomic Regions

Repetitive DNA elements present unique and persistent challenges for 3D genome analysis due to fundamental limitations in current sequencing and mapping technologies (Figure 4). These repetitive sequences, which constitute nearly half of the human genome, are notoriously difficult to analyze because most high‐throughput sequencing reads cannot be unambiguously mapped to specific genomic locations.^[^ ^82^, ^83^ ^]^ As a result, many chromatin interaction studies either exclude repetitive regions entirely or analyze them with reduced confidence, potentially missing important regulatory interactions that may be particularly relevant for stem cell biology. This analytical gap is especially concerning given that repetitive elements are frequently enriched in regulatory regions controlling pluripotency and differentiation, and many have been co‐opted as functional elements during evolution.^[^ ^81^, ^84^ ^]^ The inability to properly analyze these regions leaves significant gaps in our understanding of how 3D genome organization contributes to stem cell function and lineage commitment.

The pericentromeric and telomeric regions containing highly repetitive sequences present particularly intractable challenges for current 3D genome technologies (Figure 4). These regions form important structural elements of the nucleus and undergo dynamic reorganization during critical developmental transitions like stem cell differentiation and cellular reprogramming.^[^ ^85^, ^86^ ^]^ However, current Hi‐C protocols struggle to resolve interactions within these regions due to fundamental limitations in read mapping algorithms and the inherent sequence similarity of repetitive elements. The resulting analytical blind spots are particularly problematic because these regions are known to play important roles in nuclear architecture and chromosome positioning, which may be crucial for maintaining stem cell identity or facilitating differentiation.^[^ ^87^, ^88^ ^]^ Emerging technologies like long‐read sequencing may eventually help address some of these challenges, but significant methodological improvements will be needed before repetitive regions can be analyzed with the same confidence as unique genomic sequences.

Transposable elements present another layer of complexity for 3D genome analysis due to their abundance, sequence similarity, and dynamic regulation during development. Many transposable elements have been evolutionarily co‐opted as regulatory elements that control gene expression programs during embryogenesis and stem cell differentiation.^[^ ^89^, ^90^ ^]^ However, standard alignment tools and analysis pipelines often misassign reads originating from transposable elements or discard them as unalignable, potentially obscuring important chromatin interactions that may be mediated by these sequences. The analytical challenges are compounded by the fact that different classes of transposable elements (LINEs, SINEs, LTRs, etc.) each present unique mapping difficulties and may require specialized analytical approaches.^[^ ^82^, ^91^ ^]^ New computational approaches that specifically address repeat‐rich regions, potentially combining long‐read sequencing with specialized alignment algorithms, are urgently needed to fully characterize the 3D genome landscape and its role in stem cell biology. Until these technical challenges are overcome, our understanding of how repetitive elements contribute to chromatin architecture and gene regulation in stem cells will remain incomplete.

#### 3.1.3 Resolution and Sensitivity Limitations

Current 3D genome technologies face fundamental limitations in resolution that hinder precise mapping of chromatin interactions at the scale required to fully understand gene regulation (Figure 4). Even high‐resolution Hi‐C datasets, which require enormous sequencing depth, typically achieve resolutions no finer than 1–5 kilobases under optimal conditions.^[^ ^67^, ^92^ ^]^ This resolution gap is particularly problematic when studying small but critical genomic regions such as enhancer‐promoter interactions or boundary elements in stem cells. While precise mapping of interaction boundaries could provide crucial insights into gene regulatory mechanisms.^[^ ^93^, ^94^ ^]^ The situation becomes even more challenging when studying large genomes or complex loci where higher resolution is needed to distinguish between multiple potential interaction partners. While emerging technologies like Micro‐C and super‐resolution microscopy promise improved resolution, they come with their own limitations in throughput, cost, and technical complexity that currently restrict their widespread adoption in stem cell research.

The sensitivity of chromatin interaction detection remains another major challenge that limits our ability to study rare but biologically important interactions. Many functionally significant chromatin interactions occur at relatively low frequencies that current methods struggle to detect above background noise levels.^[^ ^75^, ^95^ ^]^ This sensitivity limitation becomes particularly apparent when studying rare cell populations, where the limited number of cells available for analysis further reduces the ability to detect weaker interactions.^[^ ^72^, ^73^ ^]^ The development of more sensitive detection methods, potentially combining molecular biology innovations with improved computational approaches for noise reduction, could reveal previously hidden aspects of 3D genome regulation. Recent advances in single‐molecule imaging and sequencing technologies may provide pathways to overcome these sensitivity limitations.

Live‐cell imaging approaches for studying 3D genome dynamics face their own set of resolution and sensitivity constraints that limit their utility in stem cell research. While providing crucial temporal information about how chromatin organization changes during processes like differentiation or reprogramming, these methods typically offer lower spatial resolution than sequencing‐based approaches.^[^ ^87^, ^96^ ^]^ Photobleaching and phototoxicity effects further constrain the duration and sensitivity of live‐cell experiments, making it particularly challenging to study delicate stem cell populations over extended time periods. The integration of super‐resolution microscopy with sequencing methods may eventually help bridge these technological gaps, but current implementations suffer from limitations in studying multiple genomic loci simultaneously.^[^ ^77^, ^88^ ^]^ For studying dynamic processes like stem cell differentiation, where both high spatial resolution and temporal information are crucial, the field still lacks robust methods that can provide comprehensive 3D genome information at the necessary scale and resolution. Overcoming these limitations will require innovations in both experimental technologies and computational analysis methods to fully capture the dynamic nature of chromatin architecture during cell fate decisions.

### 3.2 The Causality Dilemma

#### 3.2.1 Challenges in Establishing Cause–Effect Relationships Between Chromatin Dynamics and Cell Fate Decisions

A central challenge in studying the role of chromatin dynamics in stem cell fate determination is establishing definitive causal relationships between observed chromatin changes and specific lineage commitments. While numerous studies have correlated alterations in chromatin accessibility, histone modifications, and 3D genome organization with differentiation outcomes, proving that these changes directly drive cell fate decisions remains elusive.^[^ ^93^, ^97^ ^]^ For example, the opening of chromatin at enhancers associated with lineage‐specific genes often precedes transcriptional activation, suggesting a regulatory role. However, it is unclear whether these accessibility changes are causative or merely permissive for gene expression^[^ ^67^, ^77^ ^]^ (Figure 4). CRISPR‐mediated perturbations of enhancers or architectural proteins can help reveal causal relationships, but their interpretation is complicated by pleiotropic effects and compensatory mechanisms that may obscure true function.^[^ ^80^, ^81^ ^]^

The dynamic nature of chromatin further complicates causality assessments, as many modifications and structural rearrangements occur rapidly and transiently during differentiation. For instance, during hematopoietic stem cell (HSC) differentiation, widespread chromatin remodeling precedes lineage commitment, but distinguishing drivers from passengers in these global changes is challenging.^[^ ^72^, ^73^ ^]^ Single‐cell multiomics approaches have revealed substantial heterogeneity in chromatin states even within phenotypically homogeneous stem cell populations, suggesting that deterministic relationships between chromatin and fate may only emerge at the population level.^[^ ^68^, ^70^ ^]^ Additionally, the nonlinear and often stochastic nature of differentiation trajectories makes it difficult to pinpoint which chromatin changes are essential for specific outcomes versus those that are secondary consequences of another regulatory event.^[^ ^71^, ^94^ ^]^

Longitudinal studies tracking chromatin and transcriptional changes in the same cells over time are critical for resolving causality but remain technically demanding. While live‐cell imaging and sequential single‐cell sequencing methods offer promising avenues, they currently lack the resolution and throughput needed to comprehensively map chromatin dynamics during rapid fate transitions.^[^ ^87^, ^96^ ^]^ Computational modeling approaches, such as Boolean networks or dynamical systems theory, can help infer causal relationships from static snapshots of chromatin and gene expression data, but these models require validation through targeted perturbations.^[^ ^75^, ^95^ ^]^ Ultimately, establishing mechanistic links between chromatin dynamics and cell fate will require integrating genetic perturbations, time‐resolved imaging, and computational predictions to move beyond correlation.^[^ ^69^, ^81^ ^]^

Recent advances in AI‐driven computational modeling are revolutionizing our ability to decode the mechanistic relationships between chromatin dynamics and cell fate decisions. Cutting‐edge approaches now combine temporal deep learning with causal inference architectures to reconstruct the hierarchical regulatory logic governing stemness and differentiation. For instance, transformer‐based models can integrate single‐cell multiomics timelines (scATAC‐seq + scRNA‐seq) to predict how sequential transcription factor binding events drive lineage commitment,^[^ ^98^ ^]^ while diffusion models enable in silico simulation of chromatin perturbation effects for targeted epigenetic reprogramming.^[^ ^99^ ^]^ These AI systems form a closed‐loop discovery framework, then iteratively refined to establish causal, rather than correlative, design principles for stem cell control. This powerful synergy between predictive modeling and targeted perturbation finally provides the resolution needed to map the dynamic epigenetic landscape underlying cell fate transitions.

#### 3.2.2 Epigenetic Memory and Its Impact on Fate Determination

Epigenetic memory—the persistence of chromatin states inherited from a cell's developmental history—introduces another layer of complexity to the causality dilemma. Stem cells often retain epigenetic marks from previous differentiation events, which can bias their responses to subsequent fate‐determining signals.^[^ ^88^, ^100^ ^]^ For example, hematopoietic stem cells show lineage priming, with subsets exhibiting chromatin accessibility linked to specific lineages before commitment, suggesting epigenetic memory biases differentiation paths.^[^ ^72^, ^101^ ^]^ Similarly, induced pluripotent stem cells (iPSCs) frequently retain residual DNA methylation signatures from their somatic cell origins, which can influence their differentiation potential and create functional differences between iPSC lines.^[^ ^102^, ^103^ ^]^ Footprinting analysis of ATAC‐seq data can further dissect the regulatory logic of epigenetic memory—this method identifies transcription factor (TF) binding sites within specific chromatin states and quantifies their activity.^[^ ^104^ ^]^ For example, in hematopoietic stem cells, lineage‐primed regions with open chromatin often exhibit enriched footprints of key TFs, suggesting that these factors stabilize memory states through persistent binding. Additionally, comparing footprint patterns between quiescent and activated stem cells can reveal how TF competition drives memory reprogramming (e.g., replacement of HMG‐family factors by bZIP factors leading to chromatin closure).^[^ ^105^ ^]^

The mechanisms underlying epigenetic memory are diverse and context‐dependent. Histone modifications can be stably propagated through cell divisions, maintaining repressed chromatin states at key developmental genes.^[^ ^106^, ^107^ ^]^ DNA methylation provides another robust form of memory, particularly at imprinting control regions and transposable elements, where it can silence lineage‐inappropriate genes.^[^ ^83^, ^86^ ^]^ Additionally, nuclear architecture—including the spatial segregation of chromosomes into active and inactive compartments—can persist through mitosis, potentially serving as a structural memory of cell identity.^[^ ^77^, ^88^ ^]^ These memory mechanisms create hysteresis in chromatin states, where the past history of a cell influences its current regulatory landscape and future fate potential.^[^ ^108^, ^109^ ^]^

Epigenetic memory has important implications for stem cell‐based therapies, as residual marks from previous states may lead to unintended differentiation outcomes or functional impairments. In aged stem cells, accumulated epigenetic changes can restrict plasticity and reduce regenerative capacity, contributing to tissue decline.^[^ ^73^, ^91^ ^]^ Conversely, memory can also be beneficial—for example, muscle stem cells retain a “memory” of previous activation, enabling faster responses to subsequent injury.^[^ ^110^ ^]^ Understanding how to reset or preserve epigenetic memory as needed will be crucial for harnessing stem cells in regenerative medicine.^[^ ^81^, ^103^ ^]^ Experimental strategies such as prolonged culture, small molecule inhibitors, or overexpression of chromatin remodelers can help erase unwanted memory, while targeted epigenetic editing may allow for the stabilization of desirable states.^[^ ^80^, ^111^ ^]^

#### 3.2.3 Context‐Dependent Effects of Chromatin Modifiers

The role of chromatin‐modifying enzymes in fate determination is highly context‐dependent, further complicating causal interpretations. Many chromatin regulators have pleiotropic effects, acting as both activators and repressors depending on the cellular state, genomic location, and presence of cofactors.^[^ ^112^, ^113^ ^]^ For example, Polycomb repressive complex 2 (PRC2) is essential for maintaining pluripotency in ESCs but also plays critical roles in lineage restriction during differentiation.^[^ ^2^, ^114^ ^]^ Similarly, the histone demethylase KDM5B can promote or suppress differentiation depending on the stem cell type and environmental cues.^[^ ^77^, ^81^ ^]^ This context dependence makes it difficult to generalize the effects of chromatin modifiers across different systems or developmental stages.

The dynamic interplay between chromatin modifiers and transcription factors (TFs) adds another layer of complexity. TFs can recruit chromatin remodelers to specific loci, while chromatin states in turn influence TF binding, creating feedback loops that obscure causal relationships.^[^ ^90^, ^115^ ^]^ For instance, the pioneer factor FOXA1 can open compacted chromatin to enable binding of lineage‐specific TFs, but its activity depends on the pre‐existing chromatin landscape and the presence of cofactors.^[^ ^94^ ^]^ Additionally, many chromatin modifiers have noncatalytic functions—such as scaffolding protein complexes or modulating TF activity—that complicate mechanistic interpretations of loss‐of‐function experiments.^[^ ^106^, ^116^ ^]^ These multifaceted roles highlight the need for precise temporal and spatial control of chromatin modifier activity to dissect their specific contributions to fate decisions.

Technological advances are beginning to address these challenges by enabling more precise manipulation and observation of chromatin dynamics. Degron systems allow for rapid depletion of chromatin regulators, minimizing compensatory adaptations.^[^ ^81^ ^]^ CRISPR‐based epigenetic editing tools can target specific modifiers to individual loci, helping to disentangle their local versus global effects.^[^ ^80^ ^]^ Single‐cell multiomics approaches reveal how chromatin modifier activity varies across heterogeneous cell populations, providing insights into context‐specific functions.^[^ ^68^, ^70^ ^]^ Integrating these tools with computational modeling will be essential for building predictive frameworks that account for the context‐dependent nature of chromatin regulation in stem cell fate decisions.^[^ ^75^, ^95^ ^]^

### 3.3 Heterogeneity and Reversibility

#### 3.3.1 Single‐Cell Heterogeneity in Chromatin States

Stem cell populations exhibit striking heterogeneity in chromatin states, even when derived from clonal origins and maintained under uniform conditions (Figure 4). Single‐cell ATAC‐seq and Hi‐C studies have revealed diverse chromatin accessibility and interaction profiles among individual cells, reflecting a spectrum of priming states that may predispose subsets of cells to different lineage choices.^[^ ^69^, ^77^ ^]^ For example, in hematopoietic stem cells (HSCs), subpopulations with distinct chromatin accessibility patterns at myeloid‐ or lymphoid‐associated enhancers exhibit biased differentiation potential, suggesting that heterogeneity in chromatin states underlies functional diversity.^[^ ^72^, ^73^ ^]^ This intrinsic heterogeneity challenges traditional bulk assays, which average across cells and may obscure biologically meaningful variation.

The origins of chromatin heterogeneity are multifaceted, involving both deterministic and stochastic mechanisms. Asymmetric segregation of chromatin modifiers during cell division can create differences between sister cells, as observed with histone H3.3 variants in embryonic stem cells.^[^ ^117^ ^]^ Stochastic fluctuations in the expression of chromatin remodelers, such as the BAF complex, can also generate variability in accessibility across cells.^[^ ^118^ ^]^ Additionally, environmental cues—including paracrine signaling, mechanical forces, and metabolic gradients—induce context‐dependent chromatin changes that contribute to heterogeneity.^[^ ^95^ ^]^ For instance, intestinal stem cells exhibit spatially patterned chromatin states along the crypt‐villus axis, reflecting localized Wnt and BMP signaling gradients.^[^ ^94^ ^]^ These diverse sources of variation create a dynamic, heterogeneous landscape that enables flexible responses to differentiation signals.

Functional consequences of chromatin heterogeneity are context‐dependent. In some cases, heterogeneity may represent “noise” with limited biological significance, while in others, it serves as a bet‐hedging strategy to ensure population survival under variable conditions.^[^ ^84^ ^]^ For example, a subset of slowly cycling muscle stem cells retains open chromatin at self‐renewal genes, preserving regenerative capacity.^[^ ^110^ ^]^ Conversely, in cancer stem cells, heterogeneity in chromatin states may drive therapeutic resistance by enabling rapid adaptation.^[^ ^100^, ^119^ ^]^ Understanding how to distinguish functional heterogeneity from noise—and how to manipulate it for therapeutic benefit—remains a major challenge. Emerging computational tools, such as trajectory inference and RNA velocity, are helping to map the relationships between chromatin states and fate outcomes at single‐cell resolution.^[^ ^74^, ^77^ ^]^

#### 3.3.2 Plasticity and Reversibility of Chromatin States

Chromatin states are remarkably plastic during stem cell differentiation and reprogramming, but the extent and mechanisms of reversibility remain incompletely understood. During lineage commitment, widespread chromatin remodeling occurs, including the deposition of repressive marks at pluripotency loci and the opening of lineage‐specific enhancers.^[^ ^81^, ^97^ ^]^ However, many of these changes are reversible, as demonstrated by the ability of somatic cells to be reprogrammed to pluripotency.^[^ ^103^, ^108^ ^]^ The degree of reversibility varies across genomic loci: some regions, like DNA‐methylated imprints, are highly stable, while others, such as poised chromatin domains, are more labile.^[^ ^106^, ^107^ ^]^ This differential plasticity creates a “chromatin barrier” that influences the efficiency and trajectory of reprogramming.

Several factors influence chromatin reversibility, including the type of modification, genomic context, and cellular environment. Histone acetylation and DNA methylation are generally more reversible than repressive histone marks.^[^ ^117^, ^120^ ^]^ The presence of “pioneer” transcription factors, such as OCT4 and SOX2, can facilitate reversal by binding to closed chromatin and recruiting remodelers.^[^ ^75^, ^109^ ^]^ Additionally, the 3D organization of the genome affects reversibility—interactions with nuclear lamina or other structural elements can stabilize repressive states, while active chromatin hubs are more dynamic.^[^ ^88^, ^116^ ^]^ Metabolic conditions also play a role, as metabolites like α‐ketoglutarate and acetyl‐CoA serve as cofactors for chromatin‐modifying enzymes.^[^ ^121^ ^]^ Understanding these determinants is critical for developing strategies to enhance reprogramming or lock in desired differentiation states.

Therapeutic applications of chromatin plasticity face significant hurdles. While partial reprogramming approaches show promise for rejuvenating aged or damaged cells, uncontrolled reversal of chromatin states risks tumorigenesis or incomplete differentiation.^[^ ^81^, ^110^ ^]^ Epigenetic drugs, such as HDAC inhibitors or DNA methyltransferase inhibitors, can modulate plasticity but often have broad, off‐target effects.^[^ ^101^, ^122^ ^]^ More precise tools, including dCas9‐based epigenetic editors, offer targeted control but require optimization for efficiency and specificity.^[^ ^80^ ^]^ Future work must balance the benefits of plasticity—such as enhanced regenerative capacity—with the need for stability in maintaining differentiated functions.^[^ ^95^, ^120^ ^]^

#### 3.3.3 Transient States and Their Role in Fate Transitions

Stem cell differentiation and reprogramming involve transient intermediate states with unique chromatin configurations that are distinct from both the starting and endpoint populations. These intermediates are often highly heterogeneous and short‐lived, making them difficult to capture and characterize.^[^ ^76^, ^109^ ^]^ For example, during iPSC reprogramming, cells pass through a partially reprogrammed state characterized by mixed somatic and pluripotent chromatin signatures before reaching full pluripotency.^[^ ^103^, ^108^ ^]^ Similarly, hematopoietic differentiation involves a continuum of chromatin states, with gradual restriction of lineage potential rather than discrete jumps.^[^ ^72^, ^73^ ^]^ These observations challenge traditional lineage tree models and suggest that fate transitions occur through a series of metastable states (Figure 4).

The functional significance of transient states is debated. Some may represent “dead ends” where cells stall due to incomplete chromatin remodeling, while others could be necessary stepping stones that enable gradual fate transitions.^[^ ^67^, ^75^ ^]^ In muscle regeneration, a transiently activated stem cell state precedes either self‐renewal or differentiation, within chromatin accessibility changes predictive of the eventual outcome.^[^ ^110^ ^]^ In cancer, transient states with stem‐like chromatin features may facilitate metastasis or therapy resistance.^[^ ^84^, ^119^ ^]^ Distinguishing functional intermediates from noise requires high‐resolution time‐series data and functional validation—approaches that are now becoming feasible with single‐cell multiomics and live‐cell imaging.^[^ ^70^, ^77^ ^]^

Harnessing transient states could improve stem cell‐based therapies. By identifying and stabilizing specific intermediates, it may be possible to direct differentiation more efficiently or enhance reprogramming.^[^ ^81^, ^111^ ^]^ Small molecules that modulate chromatin dynamics—such as inhibitors of DNA methyltransferases—can promote transitions through bottlenecks.^[^ ^80^, ^122^ ^]^ Additionally, understanding the chromatin barriers that prevent transitions between states could inform strategies to overcome them, such as overexpression of pioneer factors or mechanical cues.^[^ ^95^ ^]^ However, therapeutically targeting transient states requires precise temporal control to avoid unintended consequences, highlighting the need for better tools to manipulate chromatin dynamics with high specificity.^[^ ^88^, ^120^ ^]^

## 4 Future Directions: From Mechanistic Insights to Precision Regulation

### 4.1 Technological Innovations for Dynamic Profiling

**Figure 5:**
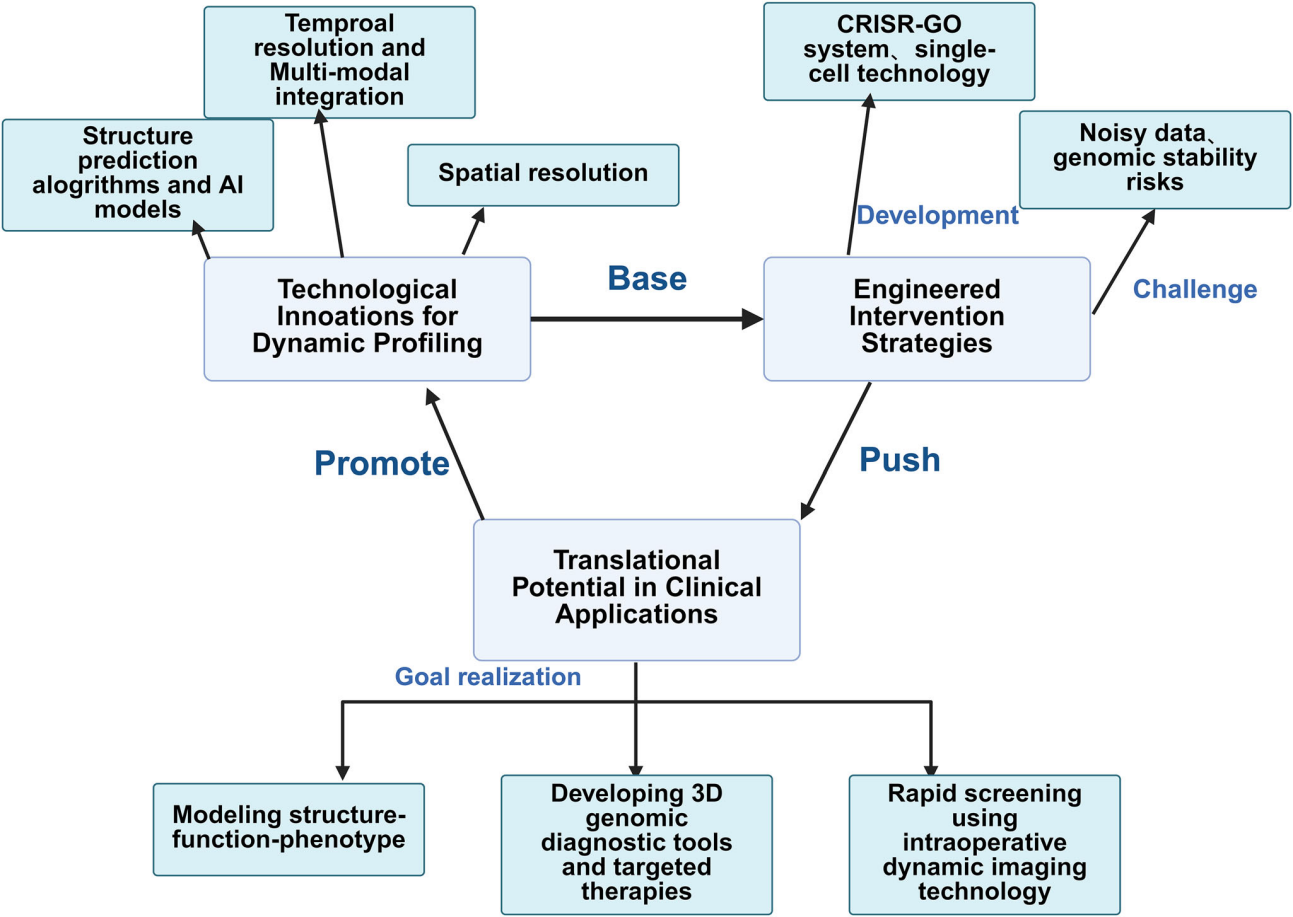
3D genome multitechnology integration to drive precision regulation. This figure presents the research framework of 3D genome. At the basic level, Micro‐C technology and optogenetic tools are utilized to provide high‐precision 3D structural data for the 3D genome. Enhance the parsing ability of 3D genomic data with the help of multimodal data integration and AI‐modeling application. Utilizing engineering intervention strategies to drive technological innovation and translation to clinical applications. The development of 3D genome is realizing from mechanistic insights to precise regulation.

As a frontier in the field of epigenetics, technological Innovations for dynamic profiling are enabling precise analysis of the dynamic evolution of 3D genome structure and function. The technological innovations in this field are mainly reflected in the following (**Figure** **5**).

#### 4.1.1 Spatial Resolution

Novel microscopic imaging technology and high‐throughput sequencing have synergistically revolutionized 3D genome resolution tools. For example, Micro‐C technology^[^ ^123^ ^]^ improves chromatin interaction resolution to the nucleosome level (about 200 bp). This advancement uniquely clarifies interactions between gene regulatory elements and mechanisms of chromatin dynamic folding. In addition, Micro‐C combined with CUT&Tag^[^ ^124^ ^]^ technology maps chromatin interactions alongside histone modifications. This approach provides a multidimensional perspective to unravel epigenetic regulatory mechanisms.

#### 4.1.2 Temporal Resolution and Multimodal Integration

A real‐time chromatin remodeling tracking system has been developed. This system is guided by optogenetic tools and combines live‐cell imaging with computational modeling. It enables the first visual reconstruction of chromatin domain folding dynamics during cell cycle progression.^[^ ^125^ ^]^ A platform has achieved breakthrough progress by integrating heterogeneous data from multiple sources (e.g., Hi‐C, ChIP‐seq and ATAC‐seq).^[^ ^23^, ^126^, ^127^, ^128^, ^129^ ^]^ This platform reconstructs dynamically changing 3D structures using time series data.^[^ ^130^ ^]^ Single‐cell multimodal generated a comprehensive dataset of the chromatin landscape of human kidney tubule cells with HiChIP,^[^ ^131^ ^]^ ChIP‐seq, and RNA‐seq.^[^ ^132^ ^]^ This study provides a valuable multiomics resource on the chromatin landscape of human kidney tubule cells. The statistical method ZipHiC^[^ ^133^ ^]^ implements a Hidden Markov Random Field (HMRF) model integrated with Approximate Bayesian Computation (ABC). It identifies both enriched interactions and experimental biases in Hi‐C datasets.

#### 4.1.3 Structure Prediction Algorithms and AI Models

Based on machine learning and deep learning, AI models are applied to chromatin 3D structure resolution. These models recognize multilayered chromatin architectures and enhance Hi‐C data^[^ ^134^ ^]^ resolution. For example, SEE^[^ ^135^ ^]^ leverages autoencoder and Transformer architectures to analyze chromatin dynamics by integrating scRNA‐seq^[^ ^136^ ^]^ data with a limited set of single‐cell Hi‐C maps. It provides a single‐cell, high‐resolution approach to dissect chromatin dynamics in developmental and disease contexts. Hier‐SSIM (Hierarchical Structural Similarity Metric) enables quantitative evaluation of chromatin topologically associating domain (TAD) hierarchies.^[^ ^137^ ^]^ This advancement provides a framework to investigate structural regulatory mechanisms underlying genome organization. Fast‐Higashi^[^ ^138^ ^]^ jointly identifies cell identities and chromatin meta‐interactions from sparse scHi‐C data. This approach helps elucidate cell‐type‐specific links between genomic structure and function. The model ChromoGen^[^ ^134^ ^]^ can predict thousands of structures in minutes, much faster than existing experimental methods of structural analysis. Using this technology, it is easier to study how the 3D organization of the genome affects gene expression patterns and function in individual cells. The model ChromInSight,^[^ ^139^ ^]^ a computational framework for systematic characterization of DSBs, resolves DNA damage response mechanisms in the context of a complex 3D genome. The first pre‐trained large model of chromatin structure, HiCFoundation^[^ ^140^ ^]^ achieves state‐of‐the‐art performance on multiple types of 3D genome analysis, including reproducibility analysis, resolution enhancement, and loop detection.

These technologies reveal transient chromatin spatial changes and establish causal links between 3D genome structure and gene regulation. They provide new perspectives for studying disease mechanisms.

### 4.2 Engineered Intervention Strategies

Engineered Intervention Strategies aim to precisely regulate gene expression and correct disease mechanisms by manipulating the 3D genome structure. Their core principle involves exploiting chromatin dynamics to repair abnormal folding patterns through tools like gene editing and spatial localization control.

#### 4.2.1 Chromatin Conformation Capture Technologies

Technologies represented by Hi‐C, Dip‐C,^[^ ^141^ ^]^ and vDip‐C, have enabled 3D genome mapping to advance from cell populations to single cells, and from common cells to rare cells (e.g., cerebellar Purkinje cells). By combining population‐scale (Pop‐C) and viral‐enriched (vDip‐C) approaches, this study achieved the first 3D genome structure resolution in single cerebellar cells^[^ ^142^ ^]^ and constructed lifespan‐spanning 3D genome atlases for both humans and mice.

#### 4.2.2 Genome Spatial Mapping and Editing Tools

The CRISPR‐Cas system directs Cas proteins (e.g., Cas9) to cleave specific DNA sequences through guide RNA (sgRNA), which triggers the cellular DNA repair mechanism to realize gene modification. CRISPR/Cas9 technology^[^ ^143^ ^]^ allows for effective gene editing of different types of cells and organisms. With these systems, homologous control of human induced pluripotent stem cells (iPSCs) is possible. The CRISPR‐Cas9 system was utilized to correct mutations in HD human induced pluripotent stem cells and generate HTT knockout cell lines, thus providing a complete set of homologous cell lines for HD research.^[^ ^144^, ^145^, ^146^ ^]^

CRISPR‐GO system^[^ ^147^ ^]^ controls genomic loci spatial localization relative to a specific nuclear region. It provides a programmable platform for studying large‐scale genome organization and function. It also physically manipulates chromatin loops or TADs to intervene in disease. This system is frequently employed to investigate large‐scale genomic organization and intervene in diseases (e.g., restoring aberrant enhancer‐promoter interactions in cancer). Being simple to use and highly efficient, CRISPR‐based genome‐editing tools are rapidly gaining popularity in biomedical research and opening up new avenues for disease treatment. Recently, pluripotent stem cell‐based, CRISPR genome engineering and forward programming to generate complex co‐culture models to study neuronal network alterations in major neurodegenerative diseases.^[^ ^148^ ^]^

Hi‐C‐derived technologies have evolved from bulk cell analyses to the single‐cell level, advancing into high‐throughput single‐cell technologies (e.g., dscHi‐C multiome). This technology simultaneously tracks transcriptomic changes and monitors dynamic remodeling during disease progression^[^ ^149^ ^]^ (e.g., intratumoral heterogeneity analysis in glioblastoma).

#### 4.2.3 Integrated Application of Sequencing and Computational Tools

The integrated application of HiFi sequencing^[^ ^150^ ^]^ and Hi‐C binning enables the generation of lineage‐resolved metagenome‐assembled genomes (MAGs) from microbial communities. This advancement propels microbial ecology and evolutionary studies, particularly in the functional profiling of gut microbiota. In plant research, combining barcode PCR with HiFi long‐read sequencing to construct a rice full protein interaction network (PPI) accelerates the study of crop functional genomics.^[^ ^151^ ^]^

Notably, such technical integration has also revolutionized cellular vulnerability analysis—for instance, DepMap's CRISPR screening relies on high‐precision sequencing (e.g., HiFi) to quantify gene knockout efficiency, while 3D genome tools like Hi‐C help decode how chromatin architecture modulates gene dependencies. By integrating Hi‐C‐derived spatial proximity data with DepMap's gene effect scores, researchers can identify context‐specific vulnerabilities: in acute myeloid leukemia, Hi‐C revealed that the HOXA9^[^ ^152^ ^]^ locus interacts with a distal enhancer, and DepMap confirmed that this regulatory loop is essential for leukemia stem cell survival, guiding the design of enhancer‐targeting differentiation therapies.^[^ ^153^, ^154^, ^155^ ^]^ This cross‐scale integration—from microbial metagenomes to cancer cell lineages—highlights how sequencing‐computational pipelines drive both basic biology and translational research, including the mechanistic understanding of cellular vulnerabilities for precision medicine.

Engineered Intervention Strategies still face technical difficulties: large data noise at single‐cell resolution, insufficient precision in vivo, and risks of genome instability from systemic 3D interventions (Figure 5).

### 4.3 Translational Potential in Clinical Applications

The translational potential in clinical applications focuses on linking chromatin spatial abnormalities to diseases and providing new dimensional biomarker targets.

#### 4.3.1 3D Genome Mapping (via Hi‐C and Single‐Cell Multiomics Techniques) and AI Models

This has identified spatial regulatory anomalies in cancers, such as topological association domain (TAD) boundary disruption and enhancer hijackin.^[^ ^156^, ^157^ ^]^ By employing 3D genome mapping, this work uncovers intratumor heterogeneity in primary glioblastoma samples.^[^ ^156^ ^]^ These findings provide novel perspectives for advancing clinical investigations into cancer biology. Studies show Lamin B1 causes enhancer‐promoter loop loss in transcription factor regions.^[^ ^158^ ^]^ This alters hematopoietic stem cell (HSC) function and fate. The application of AI models based on machine learning and deep learning greatly facilitates the study of cancer 3D genomes and further improves the regulatory map of cancer 3D genomes.

#### 4.3.2 Aging Research

Advances include consortia‐generated cell‐type‐specific^[^ ^159^ ^]^ reference epigenomes, high‐throughput DNA methylome^[^ ^160^, ^161^ ^]^ association studies. Machine learning algorithms have developed biological clocks, which provide novel insights into the mechanisms of ageing‐related diseases.^[^ ^162^ ^]^ Harvard Medical School (USA) has proposed that DNA damage‐induced chromatin modifier repositioning drives epigenetic aging.^[^ ^163^ ^]^ Various sequencing methods, such as RNA‐seq, ChIP‐seq, Hi‐C, and the ICE (inducible change of epigenome) system, support this theory.

#### 4.3.3 Mechanisms of Virus–Host Interaction

Proteins are produced by viruses during the process of infection. These proteins may participate in the transcription of host genes and change the 3D conformation of the host. These changes affect the expression and function of host genes. For example, NS1 protein of IAV can inhibit the termination of transcription, leading to sustained transcription and causing changes in the 3D structure of the host genome.^[^ ^164^ ^]^ Currently, the translational potential of clinical applications focuses on: Integrating 3D genomics with epigenetic modifications (ATAC‐seq) and protein interactions (ChIP‐seq) to construct a structure‐function‐phenotype association model. Investigating chromatin structural dysregulation in diseases. Developing anti‐cancer therapies targeting chromatin 3D structure. Creating diagnostic tools based on chromatin interaction markers using technologies such as ChIA‐PET (for example, early cancer screening). Developing intraoperative diagnostic tools based on dynamic imaging technology (Figure 5).

## 5 Conclusion

Stem cell fate decisions are shaped by the intricate interplay between spatial genome architecture and epigenetic regulation. Moving beyond static views of genome structure, recent advances reveal a dynamic, programmable genome capable of adapting to developmental and environmental cues. As 3D genomics integrates with AI, single‐cell analysis, and genome editing, it holds tremendous promise for elucidating disease mechanisms and enabling precision medicine. The future lies in translating structural insights into actionable interventions that guide cell fate, repair tissues, and combat complex diseases.

## Conflict of Interest

The authors declare no conflict of interest.

## Author Contributions

Y.H., W.L., L.L., and Y.Y. contributed equally to this work. Y.H., W.L., L.L., and Y.Y. acquired software, performed investigation, and wrote—original draft. W.S., P.L., and H.Z. wrote—review and editing. Y.L., Y.P., Q.W., Y.S., and Y.X. performed visualization. M.W. and H.Z. performed supervision. W.S., P.L., and H.Z. performed conceptualization and funding acquisition. All authors have read and approved the final manuscript.
